# Recombinant HPV16 E7 assembled into particles induces an immune response and specific tumour protection administered without adjuvant in an animal model

**DOI:** 10.1186/1479-5876-9-69

**Published:** 2011-05-18

**Authors:** Linda Petrone, Maria G Ammendolia, Armando Cesolini, Stefano Caimi, Fabiana Superti, Colomba Giorgi, Paola Di Bonito

**Affiliations:** 1 Department of Infectious Parasitic and Immune-mediated Diseases, Istituto Superiore di Sanità, Viale Regina Elena 299, 00161 Rome, Italy; 2 Department of Technology and Health, Istituto Superiore di Sanità, Viale Regina Elena 299, 00161 Rome, Italy; 3 Environment and Primary Prevention Department, Istituto Superiore di Sanità, Viale Regina Elena 299, 00161 Rome, Italy

## Abstract

**Background:**

The HPV16 E7 protein is both a tumour-specific and a tumour-rejection antigen, the ideal target for developing therapeutic vaccines for the treatment of HPV16-associated cancer and its precursor lesions. E7, which plays a key role in virus-associated carcinogenesis, contains 98 amino acids and has two finger-type structures which bind a Zn^++ ^ion. The ability of an *Escherichia coli*-produced E7-preparation, assembled into particles, to induce protective immunity against a HPV16-related tumour in the TC-1-C57BL/6 mouse tumour model, was evaluated.

**Methods:**

E7 was expressed in *E. coli*, purified via a one-step denaturing protocol and prepared as a soluble suspension state after dialysis in native buffer. The presence in the E7 preparation of particulate forms was analysed by non-reducing SDS-PAGE and negative staining electron microscopy (EM). The Zn^++ ^ion content was analysed by mass-spectrometry. Ten μg of protein per mouse was administered to groups of animals, once, twice or three times without adjuvant. The E7-specific humoral response was monitored in mice sera using an E7-based ELISA while the cell-mediated immune response was analysed in mice splenocytes with lymphoproliferation and IFN-γ ELISPOT assays. The E7 immunized mice were challenged with TC-1 tumour cells and the tumour growth monitored for two months.

**Results:**

In western blot analysis E7 appears in multimers and high molecular mass oligomers. The EM micrographs show the protein dispersed as aggregates of different shape and size. The protein appears clustered in micro-, nano-aggregates, and structured particles. Mice immunised with this protein preparation show a significant E7-specific humoral and cell-mediated immune response of mixed Th1/Th2 type. The mice are fully protected from the tumour growth after vaccination with three E7-doses of 10 μg without any added adjuvant.

**Conclusions:**

This report shows that a particulate form of HPV16 E7 is able to induce, without adjuvant, an E7-specific tumour protection in C57BL/6 mice. The protective immunity is sustained by both humoral and cell-mediated immune responses. The *E. coli*-derived HPV16 E7 assembled *in vitro *into micro- and nanoparticles represents not only a good substrate for antigen-presenting cell uptake and processing, but also a cost-effective means for the production of a new generation of HPV subunit vaccines.

## Background

Human Papillomavirus type 16 (HPV16) is associated with the development of benign and malignant lesions of the oral and genital tract [1]. The oncogenic potential of HPV16 is mainly ascribed to the viral oncoprotein E7, which has been shown to interact with a variety of cellular proteins. HPV16 E7 is a 98-amino-acid phosphoprotein (11 kDa) that binds the Zn^++ ^ion through two Cys-X-X-Cys motifs proposed to be involved in protein oligomerization [2-4]. An ATP-independent chaperone holdase activity was recently detected as the first biochemical activity of HPV16 E7 [5]. E7 is a tumour specific antigen (TSA), the mediator of tumour recognition by the host immune response [6], hence an ideal target for the development of therapeutic vaccines for treating HPV16-associated cancer and its precursor lesions [7-9].

HPV16 E7 has been expressed in various eukaryotic and prokaryotic systems [10-26] since the end of the 80s. The main objective was to produce and purify E7 in the native form to study both, its molecular structure and its cell transformation activity *in vitro*. Some of these studies have also shown the ability of E7 to form aggregates when present in high quantities. Electron microscopy micrographs of bacterial-derived E7 aggregates in particles have been shown only by Chinami *et al*. [20] and Alonso *et al*. [27]. Bacteria-derived E7 maintains the antigenic properties of the native protein, being recognised by sera from HPV infected subjects and has therefore been used in HPV serology [28-31].

The E7 protein was extensively used in vaccine development. It is a small protein poorly immunogenic (11 kDa) hence it was used with immunological adjuvants, protein and gene carriers. Various forms of therapeutic vaccines based on E7 have been developed and tested in animal models. Most of the vaccines induced E7-specific CTLs and were effective in HPV16-related tumour regression in animal models. Nevertheless, only few have reached the clinical trial phase [7-9]. As the HPV16 mouse tumour model [32] had been made available to the research community and was easy to set up, considerable work was done using E7 as antigen to demonstrate the efficacy of various adjuvants, molecular carriers and genetic vectors as inductors or enhancers of T cell response [9]. E7 has also been, fused to a number of peptides and proteins, even those of HPV16 such as L1, L2 and E6 with the aim to combine HPV prophylactic and therapeutic vaccines [6-9].

Recent progress in elucidating the cross-presentation mechanism and the role of particulate antigens in CTL immunity [33] encouraged us to use the immunogenicity of a bacterial-derived HPV16 E7, in particle form, to explore the possible development of a therapeutic vaccine against HPV16 related tumours.

This paper shows that a bacterial-derived HPV16 E7 assembles in micro- and nanoparticles on dialysis in buffer containing DTT and induces protective immunity against a tumour cell challenge in an HPV16 mouse tumour model. Interestingly, the E7 particles was administered without adjuvant. The protection of mice from tumour growth induced by the E7 particles is mediated by a strong E7-specific humoral and cell mediated immune response.

## Methods

### Protein expression and purification

Freshly streaked bacterial colonies, containing the E7 plasmid [30], were inoculated in 25 ml LB medium (DIFCO) and grown to saturation overnight (O/N) at 37°C. The culture was then inoculated in 500 ml LB, and grown until the culture density reached OD_600 _= 0.6. The His-E7 protein was induced by the addition of 1 mM IPTG (A.G. Scientific, Inc) for 3 h. The culture was harvested and centrifuged for 30 min in a Sorval centrifuge at 6000 rpm in GSA rotor. The bacterial pellet was lysed for 30 min in a rotator at room temperature (R/T) in a denaturing buffer (40 ml) containing 8 M urea (MP Biomedicals, Inc), 10 mM NaH_2_PO_4_, 10 mM Tris-HCl pH 8, 300 mM NaCl, 1 mM DTT, (Sigma-Aldrich), 1% Triton-X 114 (Sigma-Aldrich) and 1% Triton X-100 (Buffer B mod). To break the DNA, the lysate was sonicated for 60 min in the pulsed mode (50% on/off pulse; effective sonication time, 30 min) using an ultrasonic processor (Vibra-Cell 400, Sonics). The lysate was clarified in a Sorval centrifuge for 20 min at 10.000 rpm in a SS34 rotor. The supernatant was incubated for 30 min with 4 ml of 50% slurry NiNTA resin (QIAGEN) at RT. To reduce the endotoxin content, the E7-NiNTA agarose suspension was collected in a 50 ml tube, extensively washed in batch and spun down in a centrifuge at 500 × g. The E7-NiNTA was sequentially washed in Buffer B (pH 8, without detergents) containing 10% glycerol (100 ml), 20% ethanol (100 ml) and 60% isopropanol (200 ml). The isopropanol washes were alternated with cold 10 mM Tris-HCl washes (200 ml) [34]. The last sequential washes were performed using 500 ml Buffer C (8 M urea, 10 mM NaH_2_PO_4_, 10 mM Tris-HCl pH 6.3). The protein was eluted by gravity-flow in several 2 ml fractions from packed E7-Ni-NTA using 1 M Imidazole (Sigma-Aldrich) in Buffer B. After an analytical Coomassie stained SDS-PAGE, the fractions containing E7 were collected and the protein was subjected to 2 step-dialysis at 4°C in native buffers. The first step was performed in 2 L of buffer containing 25 mM Tris, 50 mM NaCl pH 7.5 (TN) in presence of 1 mM DTT and the second step was performed in 2 L of TN buffer only. E7 was concentrated in a centrifugal filter device up to a final concentration of 2 mg/ml. All the reagents were ultrapure grade. The E7 protein yield was 20 mg/l of medium culture. The protein was quantified by standard methods (Protein BC assay, BIORAD); its purity and identity were monitored by SDS-PAGE followed by Coomassie brilliant blue staining and western blotting (30). The endotoxin contamination was as low as 0.5 EU/mg protein as monitored by LAL assay (QCL-1000, Lonza). The presence of E7 particles was monitored by negative stain EM.

### SDS-PAGE and Western Blot analysis

Protein samples were separated in 12.5% polyacrylamide gels in Leammli Tris-Glycine buffer and blotted into an Immobilon-P membrane. In a non-reducing gel, the protein samples were denatured in SDS-loading buffer [30] without β-mercapto-ethanol. The protein was identified by Western blot using both commercial monoclonal and in-house prepared polyclonal anti-E7 antibodies [30]. A peroxidase-conjugate rabbit anti-mouse IgG (H+L) (Sigma-Aldrich) was used as secondary antibody. The immune complexes were revealed with a chemiluminescence substrate (PIERCE).

### Electron Microscopy Analysis

10 μl samples of the E7 preparation (2 mg/ml) were adsorbed for 1 min onto Formvar-coated copper grids, then rinsed briefly with water and negatively stained with 2% filtered aqueous sodium phosphotungstate adjusted to pH 7.0. Negatively stained preparations were observed with a Philips 208S transmission electron microscope at 80 kV.

### Zn analysis

Three samples of different E7 preparations and, as a control, three samples of Glutathione-S-transferase (GST) were analysed for their content of ^66^Zn and ^68^Zn analytical masses. The GST protein was produced in pGEX-2T transformed *E. coli *and purified by glutathione affinity chromatography (PIERCE). Measurements were performed by means of High Resolution Inductively Coupled Plasma-Mass Spectrometry (HR-ICP-MS), using an Element2 apparatus (Thermo-Finnigan, Bremen, Germany). HR-ICP-MS is a well established and powerful analytical technique for the determination of trace and ultra-trace elements in biological samples. The calibration of the method was performed by the adoption of the standard addition mode: diluted single-element standards were added to the analytical solutions. To compensate for instrumental drifts and matrix effects, indium was added to each sample as an internal standard.

### Mice immunization and tumour protection assay

6-8 week-old female C57BL/6 mice were purchased from Charles River Laboratories and maintained under pathogen-free conditions for one week before the experiment. The animal care and the experiments followed the European Directive 86/609 EEC. The protocol of animal use was evaluated by the Service for Biotechnology Animal Welfare of the Istituto Superiore di Sanità, and approved by the Italian Ministry of Health. Three groups of mice (14 per group) were inoculated subcutaneously with 1, 2 or 3 doses of 10 μg E7 respectively, at 1 week intervals. A fourth mouse group was inoculated with a saline solution and used as a control (naïve). Two weeks after the last immunization, 4 mice of each group were sacrificed to analyse the immune response and 10 mice were inoculated subcutaneously with 1 × 10^5 ^TC-1 cells/mouse, as described [35]. The TC-1 cells were grown in complete medium with 0.4 mg/ml G418. Cells at 50% confluence were harvested, counted and rinsed in Hank's medium at 1 × 10^6^cells/ml for the injection in mice. Tumour growth was monitored by visual inspection and palpation once a week for 2 months. The experiment was performed twice.

### Lymphoproliferation and IFNγ-ELISPOT assays

Splenocytes from mice of the same immunization group were pooled and enriched in CD4^+ ^and CD8^+ ^cells using the Dynal Mouse T cell Negative isolation kit (Invitrogen). Cells were cultured in RPMI 1640 (Lonza) supplemented with 10% FCS, 1% penicillin/streptomycin, 2 mM glutamine, 1 mM pyruvate and 1% non-essential amino acids (Lonza) (complete RPMI). To assess cell proliferation, the splenocyte pools (2 × 10^5 ^cells/well, in triplicate) were stimulated for five days in the presence of 5 μg/ml of two 8- and 9-mer E7 peptides, DLYCYEQL (aa 21-28) and RAHYNIVTF (aa 49-57), already known to efficiently bind the H-2 K^b ^complex of C57 Black/6 mice [36]. On day 6, the cells were pulsed with 0.5 μCi [^3^H] thymidine per well and incubated for 18 h. The cells were then harvested onto filters using an automatic harvester and counted in a Beta Counter (Wallac). The results were expressed in stimulation index (SI), calculated by dividing the mean counts per minute (cpm) of cells exposed to the E7 peptides by the mean cpm of cells incubated only with medium. The IFN-γ ELISPOT assay was performed using commercially available reagents (Mabtech AB). T-cell enriched splenocytes were seeded in triplicate (5 × 10^5 ^cells per well) in 200 μl complete medium with the E7 stimulator peptides. After 18 h at 37°C in a humidified 5% CO_2 _incubator, the plates were analysed for the presence of IFN-γ as described in [35].

### Antibody assay

The sera from each group of immunized mice were pooled and analysed. To determine the anti-E7 specific IgG titre the sera pools were serially diluted (two-fold) and assayed by ELISA [30]. The end-point dilution corresponded to an OD absorbance < 0.1 at 450 nm. Sera pools diluted 1:100 were used to analyse the anti-E7 IgM, IgA and the IgG isotypes (IgG1, IgG2b, IgG2c and IgG3). Antigen-antibody complexes were detected using the following HRP-secondary antibodies (Sigma-Aldrich): rabbit anti-mouse IgG (H+L), goat anti-mouse IgM (μ-chain), goat anti-mouse IgA (α-specific), goat anti-mouse IgG1, IgG2b, IgG3, IgG2c. HRP activity was revealed using tetramethyl benzidine substrate (TMB) in the presence of H_2_O_2_. After 30 min at RT, the enzymatic reaction was stopped by adding 50 μl of 1 M sulphuric acid/well. Washing steps were done with 400 μl/well of PBS containing 0.05% Tween-20 in an automatic washer.

### Statistical analysis

Significance analysis was performed using the Student *t *test for unpaired data. Differences were considered significant if *P *< 0.05.

## Results

### Analysis of the E7 preparation

**Figure 1:**
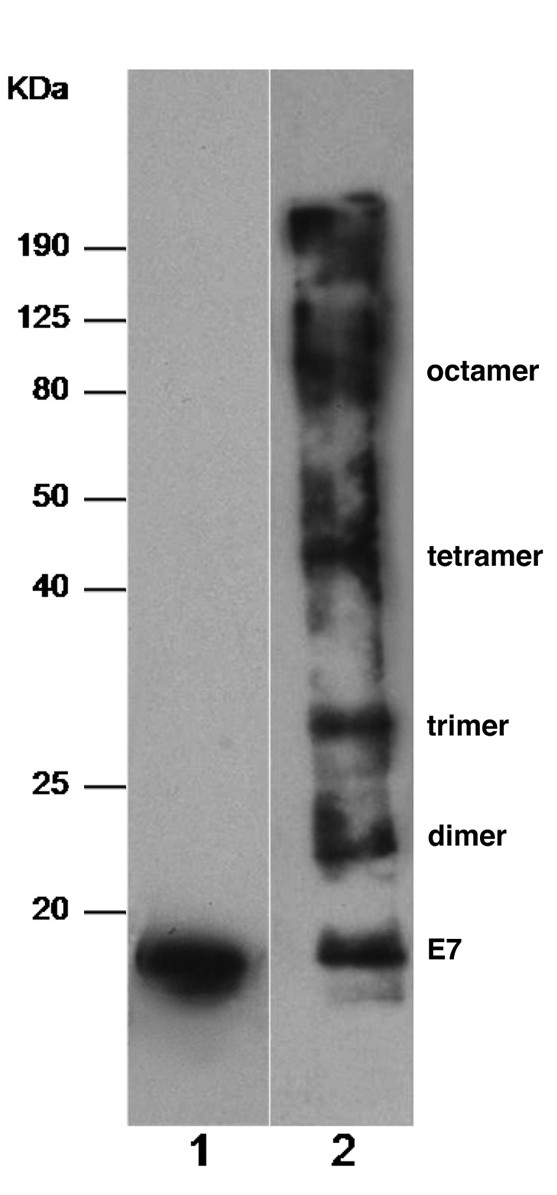
**Western blot analysis**. Western blot analysis of the HPV16 E7 produced in *E. coli *in reducing (lane 1) and non-reducing conditions (lane 2). Molecular mass markers are indicated on the left and the E7 isoforms are indicated on the right.

The E7 protein was expressed in *E. coli *with a [His]_6 _tag and purified via a one-step denaturing protocol until a high level of homogeneity and low endotoxin content were achieved [30,34]. The protein was prepared in a soluble suspension state by dialysis in Tris buffer and then analysed by western blotting in reducing and non-reducing SDS-PAGE. In the reducing gel, the E7 protein appears in monomeric form (Figure 1, lane 1). In non-reducing gels, based on the analysis of the molecular mass marker, E7 appears in forms consistent with the mass of monomer, dimer, trimer, tetramer, octamer and higher oligomers, suggesting that, in these conditions, the E7 monomer is the oligomerization unit (lane 2).

**Figure 2:**
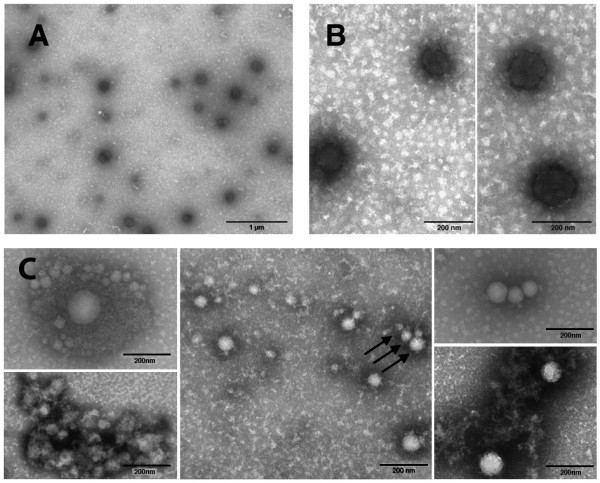
**Electron micrographs**. Electron micrographs of the negatively stained E7 preparation samples. Panel A. E7 particles of different shape and size. Panel B. Spheroidal microaggregates of compact aspect. Panel C. Highly structured E7-particles of different size are indicated by arrows. The magnitude scale bars are indicated.

Preparations of purified E7 were analysed by negative staining EM. Figure 2 shows representative EM micrographs of the E7 preparation samples. The protein appears dispersed on the grid as aggregates of different shape and size (panel A). The protein appears clustered in compact-looking spheroidal microaggregates, the majority ranging between 100 and 200 nm in size (panel B). In these same samples, E7 also appears assembled in structured particles that seem to derive from the aggregation of smaller particles (panel C). These particles resemble the previously described E7 oligomers [27]. A semi-quantitative analysis by EM counts of micro and nano-sized particles, ranging between 45-200 nm, indicates that E7-aggregates are in the order of 10^5 ^particles/ml (not shown). The E7 preparations were also subjected to EM immunolabelling but neither commercial anti-E7 monoclonal nor in-house prepared polyclonal antibodies [30] revealed any significant reaction, suggesting that these antibodies were unsuitable for the EM observation of E7-particles (data not shown).

HPV16 E7 contains a Zinc finger-like domain that binds the metal even when the protein is expressed in *E. coli *[10,11]. Since the E7 purification protocol used does not employ either chelating agents or Zinc salts, several E7 samples were analysed for Zn^++ ^content by high resolution mass spectrometry (HR-ICP-MS). The recombinant GST protein was used as a control. The Zn^++ ^ion concentration in the E7 preparations was 1.13 μg/g ± 0.10 while GST showed only trace level of Zn^++ ^(0.19 μg/g ± 0.02). The metal/protein ratio was calculated to be 0.19, therefore only about 19% of the E7 molecules were bound to Zn^++^.

### Induction of tumour-protective immunity

To investigate if this *E. coli*-derived HPV16-E7 preparation, administrated without adjuvant, was able to induce a tumour-protective immunity, groups of mice were inoculated with 10 μg of protein per mouse, 1, 2 or 3 times, at one week intervals. As a control group, mice were inoculated with a saline solution (naïve group). Two weeks after the last immunization, some of the animals were bled and killed to analyse the immune response, *in vitro*. The remaining animals were challenged with the TC-1 tumour cells and the inhibition of tumour growth in these mice was monitored for 2 months.

**Figure 3:**
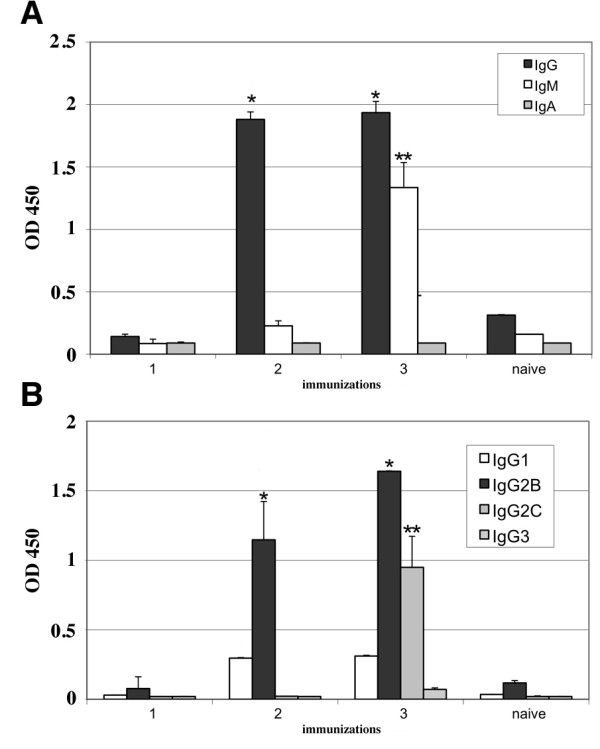
**Analysis of the antibody response**. Panel A. ELISA results showing the anti-E7 IgG (black bars), IgM (white bars) and IgA (grey bars) reactivity of the pooled mice sera samples either naïve or immunised with 1, 2 or 3 E7 doses. Panel B. ELISA results showing the anti-E7 IgG1, 2b, 2c and 3 isotype reactivity of the pooled mice sera samples.

To quantify the humoral immune response and to compare the results obtained from several animal groups, the sera from the animals of each group were pooled, then sequentially diluted to determine the antibody titres by end-point dilution in an E7-based ELISA [30]. The anti-E7 IgG titre was 1:200 after a single immunization and progressively increased in mice immunized 2 and 3 times, reaching 1:8000 and 1:16000 respectively. The presence of IgM and IgA was analysed in comparison with the IgG and the results are shown in Figure 3. In panel A, the anti-E7 specific antibodies of mice immunised 1, 2 or 3 times either with E7 or a saline solution are shown. The sera show an increase of anti-E7 specific IgG already after the second protein dose; IgMs were detected only after the third E7-dose, while IgAs were never detectable. Animals inoculated with 3 doses of saline solution did not show any E7 specific antibody response (naïve, panel A).

The therapeutic effector functions of antibodies depend on their class and subclasses [37]. In order to better evaluate the E7-specific humoral immune response, the anti-E7 specific IgG1, IgG2b, IgG2c (IgG2a) and IgG3 antibody subclasses were also determined. The results, shown in Figure 3, panel B, show that the IgG2b level was significant after the second immunization while the level of IgG2c was significant only after the third immunization. The level of IgG1 was not significant and IgG3s were undetectable. This anti-E7 IgG isotype profile indicates that the immune response induced in vaccinated mice is a mixed Th1/Th2 type.

**Figure 4:**
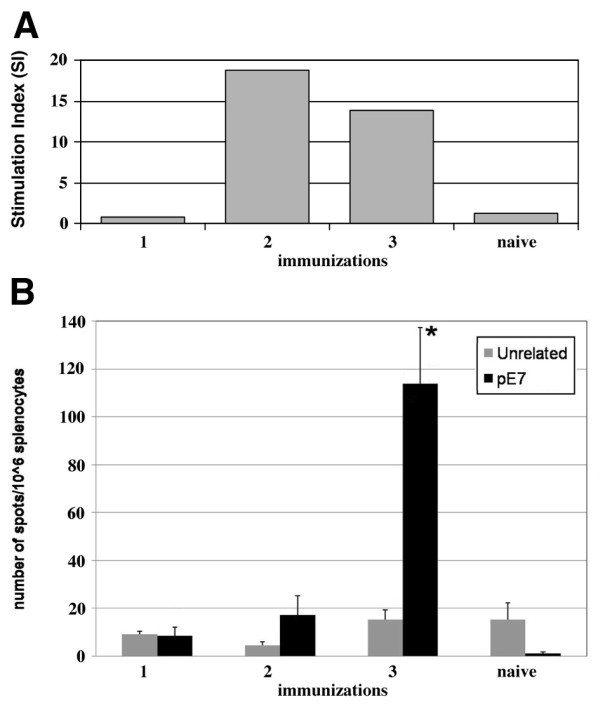
**Analysis of the cell-mediated immune response**. Panel A. T cell proliferative response of C57BL/6 naïve and mice immunised with 1, 2 o 3 E7 doses. Panel B. IFN γ- secreting cells from naïve mice and mice immunised with 1, 2 o 3 E7 doses. Cells were stimulated with two CTL E7 peptides (black bars) or with an unrelated peptide (grey bars).

To analyse the induction of the cell-mediated immune response in mice after 1, 2 or 3 doses of the E7 preparation, T-enriched splenocytes from mice of the same immunization group were stimulated *in vitro*, with the E7-specific CTL peptides and processed for T cell proliferation and γ-IFN ELISPOT assays. Splenocytes from naïve mice were pulsed with an unrelated mixture of peptides used as control. The results are shown in Figure 4. Splenocytes from mice immunised with 2 and 3 doses of E7 showed a high Stimulation Index (SI) suggesting that specific T clone selection occurred after E7 peptide stimulation. Splenocytes from naïve mice and from mice immunised once showed non-significant SI. Conversely, in the γ-IFN ELISPOT assay (panel B) only the splenocytes of mice that received 3 E7 doses, stimulated with the E7-specific CTL peptides, showed a significant level of E7-specific γ-IFN producing cells (panel B, 3).

**Figure 5:**
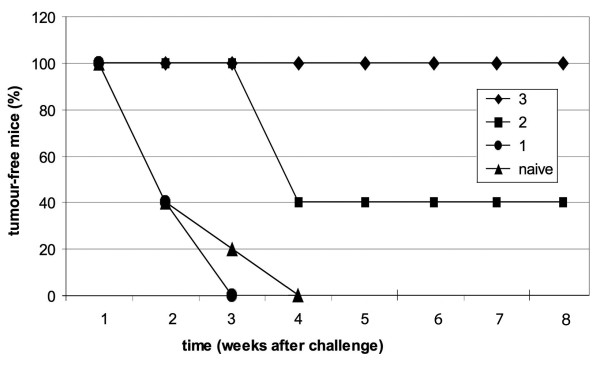
**Tumour protection experiment**. Mice either naïve or vaccinated with 1, 2 and 3 doses of E7 were challenged with 1 × 10^5 ^TC-1 tumour cells and the tumour growth was monitored weekly. The percentages of mice without tumour are indicated.

To evaluate the efficacy of the E7 preparation as inductor of anti-tumour immunity, the mice immunised with 1, 2 or 3 doses of E7 were challenged with the TC-1 tumour cells, and the tumour growth was monitored for two months after the challenge. The results are shown in Figure 5. Mice vaccinated with three doses of E7 particles were fully protected from tumour growth. Only 40% of the mice immunised with 2 doses of E7 were tumour free, whereas the mice immunised with 1 dose and the naïve mice developed a palpable tumour within 4 weeks of tumour-monitoring, after the challenge with TC-1 cells.

## Discussion

This study reports the induction in mice of a tumour-protective immunity using an *E. coli*-derived HPV-E7 preparation containing particles. E7 has been intensively studied for many years. However, this is the first time, to our knowledge, that a particulate form of E7 has been used as an immunogen and proposed as a non-adjuvated vaccine. Our results show that, the tumour-protective immunity in the mouse TC1/C57BL/6 tumour model correlates to the elicited E7-specific T cell response, and to the IgG isotype switching (IgG2b and IgG2c).

Previous studies on bacterial-derived E7 showed that Zinc has a role in E7 particle formation. Chinami *et al*. [20] obtained E7 nanoparticles using Zinc acetate both in culture medium and purification buffers. On the contrary, Alonso *et al*. [27] obtained well defined-E7 oligomers after EDTA chelation of Zinc. For our E7 preparations, neither the culture medium nor the purification buffers contained Zinc salts. The analysis of the Zinc content in our protein preparation indicates that only 19% of E7 binds the metal, suggesting that several forms of particles could be generated from the bacterial-derived E7. The metal does not seem important for the formation of our E7 micro- and nanoparticles, at least not in the experimental conditions used here. We did not increase the Zn^++ ^content in the E7 preparation used as immunogen in mice, considering that while Zn^++ ^is an essential mineral in eukaryotic systems, a high quantity of the metal is also toxic [38]. When Zinc was removed from the E7 preparation by dialysis in the presence of 1 mM EDTA, the protein's solubility decreased resulting in salting out of E7 as large aggregates without forming micro- and nanoparticles, as observed by EM (data not shown).

As the aim was to obtain a highly immunogenic E7 preparation, we did not focus on obtaining identical particles, considering that particles of different size can be taken up by different types of antigen presenting cells, such as dendritic cells, macrophages and polymorphonuclear leukocytes, sustaining a more potent immune response [39,40]. However, we standardized the different preparations by semi-quantitative counting of particles on EM micrographs (not shown).

The immunogenicity of *E. coli*-derived E7 fused, through the N-terminus, to either HPV16 E6 or GST, was also investigated in mice. An antigen-specific immune response of Th2 polarity was obtained when the fusion proteins were administered to mice without adjuvant (data not shown). However, we were unable to observe the typical micro- and nanoparticles in these E7-fusion proteins prepared from *E. coli *(data not shown).

Recently, the cytosolic accumulation of E7-oligomers shown in HPV16 cervical cancer cell lines and in clinical samples by indirect methods, supports a new hypothesis regarding the presence of E7 isoforms and their role in different cell compartments [41-43]. As keratinocytes display antigen-presenting cell features [44], the presence of E7 in different aggregation forms and cell compartments could affect E7 processing and presentation by MHC I and II molecules, determining both the strength and quality of the host's anti-HPV immunity.

More studies on recombinant *E. coli*-derived E7, assembled in different forms, would contribute to explaining how the different branches of the immune system in the HPV16 mouse tumour model are stimulated. Significant differences exist between the HPV16 mouse tumour model and human HPV16-dependent diseases. However, studies on IgG subclasses and their FcγR receptors between mouse and human are comparable (37). We believe that HPV16 E7 immunogenicity studies in mouse will provide insights into the understanding of the protective immunity against human HPV16 infections as well.

The commercial preventive HPV vaccines have high production costs which has made widespread vaccination programs still not possible. Recently, new combined preventive and therapeutic HPV vaccines produced in *E. coli *have been described [45-47] and the data presented here suggests a possible use of *E. coli*-derived E7 in particle form in subunit vaccines. The *E. coli *expressed proteins represent a well-studied and cost-effective means for the production of vaccines. These methods require reduced time, costs, labour and can be easily scaled up in industrial-scale productions. A generation of new low-cost HPV vaccines could represent the only possibility for women living in developing countries to gain access to HPV vaccination programs to prevent or treat pre-cancerous lesions and cancer.

## Conclusions

The paper describes, for the first time, the use of recombinant HPV16 E7, assembled *in vitro *into particulate form, to induce protective immunity against a HPV16-related tumour in an HPV16 mouse tumour model. Data show that E7 particles, used without adjuvant, are excellent stimulators of the immune system. In C57BL/6 mice, the E7 preparation induces anti-tumour immunity sustained by both humoral and cell-mediated immune responses. This E7 protein (derived from *Escherichia coli*) without adjuvant could represent, along with the recently proposed *E. coli*-derived HPV antigens [45-47], a low cost constituent for the development of a new generation of HPV16 vaccines, which combine prophylactic and therapeutic activities.

## Competing interest

The authors declare that they have no competing interests.

## Authors' contributions

LP carried out the biochemical and immunological assays, made contribution to the analysis and interpretation of the data and helped to draft the manuscript; MGA carried out the EM analysis and made contribution in data analysis; AC performed the experiments with the animals. SC carried out the mass spectrometry experiments; FB made contribution in data analysis; CG made contribution to the analysis and interpretation of the data, in critical revision of the manuscript and in acquisition of funding. PDB conceived and designed the study, analysed and interpreted the data and drafted the manuscript. All authors read and approved the final manuscript.

## Authors' information

LP's present address: Istituto Nazionale Malattie Infettive "L. Spallanzani", Rome. CG 's present e-mail: ros.giorgi@gmail.com.
